# Evaluating the internal structure validity of admissions measures: do they capture distinct attributes?

**DOI:** 10.3389/fvets.2026.1814101

**Published:** 2026-07-22

**Authors:** Kent G. Hecker, Sneha Shankar, Connor Maxey, Rob McCorkell

**Affiliations:** 1 Faculty of Veterinary Medicine, University of Calgary, Calgary, AB, Canada; 2 Faculty of Medicine and Health Sciences, McGill University, Montreal, QC, Canada

**Keywords:** admissions, Exploratory Factor Analysis, internal structure validity, multiple attributes, validation

## Abstract

**Introduction:**

Veterinary admissions committees aim to select applicants who will successfully complete a Doctor of Veterinary Medicine (DVM) program and contribute effectively to the profession. Although traditional admission procedures emphasize academic indicators such as undergraduate grade point average (uGPA) and standardized test scores, increasing attention has been placed on assessing social–emotional skills and school-specific contextual attributes. Given the high-stakes nature of admissions and the claim that multiple attributes are assessed at the time of selection, internal structure validity evidence is required to support the use of scores from admissions measures in the selection process. This study examines whether commonly used admissions measures reflect distinct academic, social–emotional, and contextual factors.

**Methods:**

Participants included 338 applicants (78.17% female) in a retrospective cohort study from the 2022–2024 admissions cycles at the Faculty of Veterinary Medicine, University of Calgary, Canada. Admissions measures included uGPA, Medical College Admissions Test (MCAT) total and subscores, Multiple Mini-Interview (MMI) scores, Computer-based Assessment for Sampling Personal Characteristics (Casper) situational judgement scores, standardized essay ratings, and structured file review scores. Bivariate correlation and Exploratory Factor Analysis (EFA) were used to explore the relationships and internal structure between the scores from these admissions measures.

**Results:**

Correlations among admissions variables showed small to moderate associations, with expected clustering among academic indicators. EFA produced a three-factor solution accounting for 53.36% of the variance which were labeled: (1) Academic Ability (uGPA; MCAT academic subscores), (2) social– emotional skills (essay, Casper, MCAT social emotional sub score; partial MMI loading), and (3) contextual factors (file review; partial MMI loading).

**Discussion:**

Findings provide internal structure validity evidence supporting the claim that admissions measures capture multiple attributes and these measures should be considered collectively as a system of assessment. Notably, the MMI contributed to both social–emotional and contextual factors, highlighting its multifaceted nature. These findings provide a foundation for longitudinal research examining the predictive validity of academic, social–emotional, and contextual factors for performance throughout veterinary training.

## 1 Introduction

Veterinary admissions committees are charged with selecting applicants that demonstrate the traits necessary to successfully complete a veterinary program with the intent to contributing meaningfully to the profession (1–3). Traditional models of applicant selection for admission to veterinary programs have emphasized objective measures of academic, or ‘cognitive’ factors, such as cumulative undergraduate and science grade point average (uGPA), and standardized test scores [i.e., Graduate Record Examination (GRE), Medical College Aptitude Test (MCAT), Veterinary College Aptitude Test (VCAT)]. There is widespread acknowledgment, however, that current admissions models must extend beyond assessment of academic factors to include ‘social–emotional skills’ (often times referred to as non-cognitive or non-academic factors) into selection, to reflect the diverse attributes of competent veterinarians (4–7). Further, there are contextual factors associated with schools that admissions committees may take in to consideration. These contextual factors may include, but are limited to: Is there a rural mandate? Do schools track for various species? Is there an emphasis on public health? Use of all of these requirements are incorporated in so called holistic or mission driven admissions processes (8, 9). However, given the high stakes nature of admissions processes, validity evidence is required to support the use and claims of various assessment methods at the time of admissions. There are multiple sources of validity evidence which could include: (a) content, (b) response process, (c) internal structure, (d) relation to other variables, and (e) consequences (10). One source of validity evidence based on internal structure, aids in substantiating/supporting the claims/decisions made regarding the relationship between items, subscales, and total scores within and across assessment methods (10–12). This study, therefore, explores the internal structure validity evidence of admissions measures that have been reported to measure multiple constructs (attributes) at the time of selection.

As indicated above, in North America, all veterinary schools use a combination of academic measures. This typically includes grade point average (uGPA) performance on pre-requisite courses and/or overall academic performance. How they are used to select applicants is not standardized across schools. uGPA measures may be used as a threshold to select applicants for the next phase of an admissions process (such as an interview process) and then not used in a final admissions calculations or used to select for the next phase and also weighted in a final admissions calculation, or any number of combinations. The assumption is that the pre-matriculation uGPA from across a number of institutions are comparable and reliable (13, 14). However, this assumption has not been well reported within the veterinary medicine literature (15). The predictive validity of uGPA for future performance in health professions programs are typically reported as correlations to future performance in pre-clinical and clinical years (13, 16). Standardized exams such as the GRE, MCAT and VCAT have been used intermittently in veterinary medicine (17–19). Given the nature of development and standardization of these assessments, the reliability of these standardized measures are well documented and the relationships with future performance is typically stronger than use of uGPA measures (18, 20–23). In other words, standardized total scores and their subscores from these standardized measures are stronger indicators for future academic performance with uGPA providing incremental validity. However the use of standardized assessments have been called into question in veterinary medicine prompting calls for stronger and more transparent evidence regarding their fairness, and appropriate use (24).

Recently, more emphasis has been placed on other character attributes, or social–emotional skills, that contribute to selection of those admitted. While academic ability have been found to correlate with performance in medical and veterinary education, social–emotional attributes are receiving increased attention in the research (7, 25, 26). The shortlist of social–emotional factors that could be assessed at the time of admissions is debatable, but admission committees generally consider professionalism, reasoning, empathy, communication skills, and interpersonal skills as important social–emotional skills (27, 28). Schools have typically attempted to assess social–emotional factors with autobiographical statements, letters of reference, essays, and/or interviews plus standardized assessments like Computer-based Assessment for Sampling Personal Characteristics (Casper) and the Critical Analysis and Reasoning Skills (CARS) subscore of the MCAT (29). Early investigations of admissions processes used for social–emotional skills in medicine and veterinary medicine have provided preliminary evidence for the reliability and validity of MMI scores (25, 27, 30, 31) and convergent/divergent validity for scores from a standardized proctored essay component (32). Casper is an online situational judgement vignette assessment of non-academic competencies which include but are not limited to, collaboration, communication, professionalism empathy and equity (33, 34). There are reported studies for the construct validity of scores to assess future licensure performance (34), evidence from veterinary medicine is limited and mixed for its use and interpretation (35). The reliability and validity of the new MCAT total score and subscores has not yet been reported in veterinary medicine. Predictive validity evidence has been reported in medicine and osteopathic medicine (23, 36). There is still a gap in the validity literature regarding the claims that schools are selecting for these factors at the time of admissions let alone how they relate to future performance in the same domains in pre-clinical and clinical years (26, 37, 38).

School specific components or contextual factors have not typically been reported. File reviews, personal selection, and even interviews, could be considered proxy measures not only of social and emotional attributes but of contextual factors from the respective schools. Validity evidence of the contextual attributes, viewed through this lens, appears to be less apparent in the literature. Indeed, work looking at the reliability and predictive validity of scores from personal statements have repeatedly shown that content and type of personal statement provided is not predicative of future performance in health professions schools (39, 40). Although there are claims that these statements and demographics are predictive of the type of practice that graduating veterinarians first choose (41, 42). Therefore, work looking at values may be tapping into these contextual factors (43).

Given the claims that admissions processes select for more than academic ability and to justify the use of time- and resource-intensive selection factors, such as interviews and standardized assessments, ongoing validity evidence is required—all prerequisites of best practice (44). As admissions processes look at a diverse array of factors for selection purposes, internal validity evidence, which examines the relationship between variables to support use of scores in decision making purposes, is necessary (11). Therefore, the objective of this study is to examine the internal structure validity evidence of admissions data to evaluate the claim that multiple attributes (such as academic ability, social–emotional skills, and others) are measured during the admissions process.

## 2 Methods

This is a retrospective cohort study of 3 application cycles at a large, western Canadian University, University of Calgary, Faculty of Veterinary Medicine (UCVM) in the Doctor of Veterinary Medicine (DVM) program. Applicants must be residents of the province of Alberta where the University is located. This study was reviewed and was deemed exempt from the University of Calgary Conjoint Faculties Research Ethics Board.

### 2.1 Participants

Three hundred and thirty-eight applicants (78.17% female) in the 2022 (*n* = 94), 2023 (*n* = 124) and 2024 (*n* = 120) admissions cycle for the DVM program are included in this study.

### 2.2 Variables

#### 2.2.1 Undergraduate academic performance

Grades from the last 2 years of full-time undergraduate coursework (as identified by individual applicants) and from 10 required courses are required to apply. University grades were converted to the standard 4-point GPA scale. The minimum academic criteria include a passing grade in 10 required courses (two biology, two chemistry, one English, one organic chemistry, one statistic, one biochemistry, one genetics, and one ecology course). Given this criteria for the 10 required courses (a dichotomous decision, pass or did not pass), GPA from the required courses is not used in further analyses. A minimum of a 3.0 (on a 4.0 scale) from the four most recent semesters with a full course load is required (uGPA, defined as minimum of four ‘first-attempt’ courses per semester taken between the months of September—December and January—April). This uGPA measure is used for all following analysis.

#### 2.2.2 Medical College Admissions Test (MCAT)

Total MCAT score and MCAT subscores [Chemical and Physical Foundations of Biological Systems (CPBS), Biological and Biochemical Foundations of Living Systems (BBFL), Psychological, Social, and Biological Foundations of Behavior (PSBB), Critical Analysis and Reasoning Skills (CARS)] were collected and used for this study.

#### 2.2.3 Multiple-Mini Interview (MMI)

The MMI comprised a series of seven eight-minute interview stations. There are typically two raters per station, one veterinarian and one UCVM faculty members. Stations consisted of situational events where applicants were asked to consider an issue intended to assess one of seven attributes/competencies which are reviewed annually. These could include but are not limited to knowledge of veterinary medicine, moral and ethical reasoning, economics (value of life), teamwork (interpersonal skills), empathy, career adaptability, and applicability of the candidate for the UCVM DVM program.

Two trained raters independently assess the attributes of applicants with at least three items on a 5-point scale (1 = unacceptable to 5 = exceptional). To compare MMI scores across years, *Z*-scores were created within year. A *Z*-score was computed per station, and the total *Z* score from across the stations by year was calculated. Therefore, the standard deviation for the MMI-scores is greater than the standard SD of 1. These *Z*-scores are used within the analyses.

#### 2.2.4 Computer-based Assessment for Sampling Personal Characteristics (Casper)

A situational judgement test, administered online where participants respond to various scenarios meant to assess various social–emotional skills. *Z*-scores provided by Acuity Insights were used in these analyses (45).

#### 2.2.5 Essay

The essay component was 1 h long, with an option to write 750 words on the specified topic by hand or via computer. Each essay was converted to an electronic format and was rated independently by three trained raters using a standardized rubric. Written communication skills and specific understanding of the essay topic using 5-point Likert-type scales. Given the essay and number of items may change by year essay scores within year were converted to a *Z*-score in order to combine data across years. Data regarding the validity of the scores are presented in Hecker and Violato (32)

#### 2.2.6 File review

Applicants submit to a structured file review, where applicants outline their interests and related experience in veterinary medicine (Production Animal Health, Ecosystem and Public Health, Equine Health, Investigative Medicine, Small or Companion Animal, General Veterinary Practice and other), their academic honours, their work experience, extracurricular activities and their volunteer and community activities. These file reviews were scored by 3 independent raters to assess their applicability and suitability for the UCVM DVM program.

### 2.3 Admissions procedure

Selection for a Multiple Mini Interview (MMI) is based upon a threshold of academic performance from the four (4) full-time term uGPA of >3.0, and a Score of >495 on the MCAT. During this time period, there were a total of 371 applicants, of which 338 (91%) had complete data for this study. The 33 applicants not included in these data either did not meet the GPA or MCAT requirement or did not have complete data across the other measures. Applications meeting or exceeding the threshold are then subject to a file review and must complete Casper. The file review process includes both objective and subjective assessment of non-academic attributes by three (3) raters. Data are deidentified, the raters are not provided uGPA scores, MCAT scores, sex, age, and name of the applicant. Applicants selected for interviews are also required to sit for an hour-long proctored essay. Essays are assessed by 3 raters using a standardized scoring rubric. Final admissions scores are based on MMI score—65%, Casper score—5% and Essay score-30%.

### 2.4 Analyses

To assess the internal structure validity evidence to support the claim that the admissions criteria are measuring more than academic ability the following analyses were performed. First, descriptive statistics and bivariate correlations (Pearson’s *r*) were calculated amongst admissions variables. Given the sample size (*n* = 338) and the distributions of the data (data were checked for skewness, kurtosis), we proceeded as the data were pseudo continuous. Specifically, reliability analyses (Generalizability theory and Cronbach’s alpha) are reported where they could be calculated. Generalizability analyses of the MMI consisted of a partially nested design with participants, stations, and rater nested within station. Cronbach’s alpha was used to report on internal consistency for the file review and essay scores. Exploratory Factor Analysis (EFA) was then performed after determining if the Kaiser–Meyer–Olkin (KMO) measure of sampling adequacy and Bartlett’ test of sphericity met the criteria to test if the correlation matrix is factorable. EFA proceeded using a principal components analysis with varimax rotation to determine the underlying factor structure of the admissions variables using SPSS (46). The cut-off for factor loadings was set to .40.

## 3 Results

Table 1 summarizes the descriptive statistics and correlations for all the described admissions variables. For these variables skewness ranged from −1.05 (uGPA which is expected) to 0.70. Kurtosis ranged from −0.789 to.864. These are all within acceptable ranges to proceed with the following analyses. The correlations show small to medium effects sizes amongst the academic scores, from the MCAT subscores and uGPA. The correlations found between MMI scores and file review (*r* = 0.27), CARS (0.19) and Casper (0.32) scores indicate further analyses could elucidate how these variables inter-relate. The reliability for the MMI (*G*-coefficient) was 0.75 for the 2022 admissions cycle, 0.79 for the 2023 admissions cycle and 0.78 for the 2024 admissions cycle. The reliability for the file review (coefficient alpha) was 0.66 for the 2022 admissions cycle, 0.48 for the 2023 admissions cycle and 0.50 for the 2024 admissions cycle, and the essay was 0.88 for the 2022 admissions cycle, 0.76 for the 2023 admissions cycle and 0.91 for the 2024 admissions cycle.

**Table 1:** Descriptive statistics [mean and standard deviation (SD)] and correlations for all admissions variables (*n* = 338).

Admissions Variables	uGPA	CPBS	CARS	BBFL	PSBB	File_review	Casper	MMI_zscore	Mean (SD)
uGPA									3.70 (0.24)
CPBS	0.30**								124.86 (1.91)
CARS	0.09	0.09							125.23 (2.01)
BBFL	0.32**	0.64**	0.18**						125.77 (1.99)
PSBB	0.20**	0.34**	0.29**	0.44**					126.30 (2.14)
File_review	−0.02	0.00	0.02	0.02	−0.01				10.35 (3.39)
Casper	0.03	0.07	0.07	0.01	−0.02	0.02			0.75 (0.83)
MMI_zscore	0.08	0.00	0.19**	0.09	0.15**	0.27**	0.32**		0.01 (4.63)
Essay_zscore	0.00	0.12*	0.10	0.10	0.10	0.03	0.10	0.11*	0.01 (1.00)

^**^p < 0.01, ^*^p < 0.05, uGPA, undergraduate grade point average; CPBS, Chemical and Physical Foundations of Biological Systems; CARS, Critical Analysis and Reasoning Skills; BBFL, Biological and Biochemical Foundations of Living Systems; PSBB, Psychological, Social, and Biological Foundations of Behavior; Casper, Computer-based Assessment for Sampling Personal characteristics; MMI, Multiple Mini Interview.

Table 2 shows the results from the EFA. The KMO for the data is 0.640 and the Bartlett’s test of sphericity is *p* < 0.001. With a high KMO > 0.60 and a significant Barlett’s test the correlation matrix is therefore factorable. A 3 factor solution for the EFA (accounting for 53.36% of the variance) was determined. uGPA, and the MCAT subscores CPBS, BBFL and PSBB, loaded on the first factor labelled “academic ability.” Essay, Casper, MMI (split loading) and the MCAT CARS subscore loaded on the 2nd factor, “social emotional skills.” File review scores and MMI (split loading) loaded on the third factor, “UCVM contextual factors.”

**Table 2:** Exploratory Factor Analysis of admissions measures indicating a three factor solution labeled academic ability, social emotional skills and contextual factors.

Admissions Variables	Academic ability	Social and emotional skills	Contextual factors
uGPA	0.58		
CPBS	0.80		
CARS		0.55	
BBFL	0.85		
PSBB	0.64		
File_review			0.82
CASPer		0.56	
MMI_zscore		0.45	0.68
Essay_zscore		0.63	
Variance accounted for	25.79	16.24	11.35

uGPA, undergraduate grade point average; CPBS, Chemical and Physical Foundations of Biological Systems; CARS, Critical Analysis and Reasoning Skills; BBFL, Biological and Biochemical Foundations of Living Systems; PSBB, Psychological, Social, and Biological Foundations of Behavior; Casper, Computer-based Assessment for Sampling Personal Characteristics; MMI, Multiple Mini Interview.

## 4 Discussion

The use of various selection processes used in admissions is based on the claim that the processes are measuring multiple attributes of applicants in a system of assessment that will ensure the greatest likelihood of success in health professions program. Given the high stakes nature of selection processes for veterinary schools, internal structure validity evidence is required to support the claims the methods are selecting different fundamental attributes. These attributes are typically academic ability, social emotional skills, and potentially contextual factors that are unique to the program. These attributes (also referred to as factors, or latent constructs as they are not directly measured) are assessed by several methods that theoretically measure components of these attributes. The main findings of our work are: 1. There are small to moderate correlations between admissions score measures suggesting the methods used are measuring different attributes: and 2. The EFA produced a three-factor solution accounting for 53.36% of the variance which were labeled: (1) academic ability (uGPA; MCAT academic subscores), (2) social–emotional skills (essay, Casper, MCAT social emotional sub score; partial MMI loading), and (3) contextual factors (file review; partial MMI loading). This work provides sources of validity evidence to support the use of the selection methods for these underlying attributes that are used for selection criteria for this specific school. In this case, using EFA there is internal structure validity evidence for combining scores from measures that are reported to assess academic ability, social–emotional skills, and context specific factors that veterinary admissions committee use as a system of assessment to make decisions for admissions.

The measure for academic ability for most veterinary schools has been pre-matriculation uGPA scores, whether from required courses, the last 4 semesters, or a combination of both uGPA measures. The MCAT (and other standardized tests) has been used sporadically by veterinary schools for admissions processes. Here we show that the subscores for Chemical and Physical Foundations of Biological Systems [CPBS], Biological and Biochemical Foundations of Living Systems [BBFL], Psychological, Social, and Biological Foundations of Behavior [PSBB] and uGPA are related and measure what we labelled “academic ability.” Over the last two decades, there has been an increased awareness of and a push to select for social and emotional skills (or non-cognitive factors), however, the reliability and validity of scores from these methods have been mixed (14, 47). Here we show that the scores from Critical Analysis and Reasoning Skills [CARS-MCAT subscore], the MMI, a standardized essay, and Casper appear to be measuring the same underlying latent attribute that correspond to this attribute. Reviews in medical education consistently show that scores from academic ability, MMIs, and situated judgement tests, like Casper, tend to relate to different aspects of future performance and often exhibit modest intercorrelations, reinforcing a *both/and* rather than *either/or* framing (14). Within veterinary admissions, prior research indicates that pre-matriculation uGPA and standardized tests (e.g., GRE verbal) relate to later academic assessments (e.g., VEA, NAVLE), while interpersonal/behavioral assessments relate better to clinical and professional outcomes (17, 19, 48, 49).

The finding that scores from the personal statement (file review scores) and MMI (split loading) load on a separate factor (here we label as school specific “contextual” factor) is intriguing to consider. While previous work has shown that scores from personal statements (especially written off site) are not reliable nor predictive for future performance (40), could there be components in the scores from these measures that reflect what raters think (regardless of training) that reflect the contextual factors of the school and an applicant’s fit to these contextual factors? Could this be a measure that raters believe assist with success in the veterinary program? Or, is this a measure of bias which may affect future performance of selected applicants in the program? Future research should examine rater cognition while scoring, and applicant intentions when producing, personal statements to better understand what these tools measure—and whether they measure school specific factors or introduce systematic bias into the selection process.

This investigation was conducted at a single veterinary program, potentially limiting generalizability. Nonetheless, the admission tools examined—academic metrics, social-emotional assessments, and contextual measures—reflect practices used widely across veterinary and other health professions programs, suggesting broader relevance (14). Generalizations regarding the validity of selection tools should be made cautiously. The essay and MMI are resource intensive, so their utility and feasibility warrant consideration. The use of standardized assessments such as the Casper and MCAT come at a cost that may exclude some candidates from applying. However, given the current context where a passing grade is required for the required courses and a minimum uGPA is required for the last two full time semesters, we argue taking the MCAT is less cost prohibitive than spending another semester at an undergraduate program taking a Biochemistry pre-requisite again (as an example), paying tuition, and room and board. With the evidence of moderate reliability and internal consistency validity, organizations will have to make their own judgement whether these improvements and use of these methods are worth the cost.

## 5 Conclusion

This study provides internal structure validity evidence for a veterinary admissions process comprising academic metrics, social-emotional assessments, and contextual measures, yielding a three-factor solution. These findings offer a baseline for subsequent predictive validity studies. Future work should link the identified factors to specific training outcomes across cognitive, psychomotor, and affective domains, evaluate fairness, and track resources. By systematically studying and extending the validity argument for the use (or not) of these measures, veterinary programs can refine weighting and design of admissions tools to make defensible, fair, and efficient selection decisions.

## Data Availability

The data analyzed in this study is subject to the following licenses/restrictions: due to the sensitive nature of the data, these data are not available. Requests to access these datasets should be directed to kghecker@ucalgary.ca.
